# First Discovery of Phenuiviruses within Diverse RNA Viromes of Asiatic Toad (*Bufo gargarizans*) by Metagenomics Sequencing

**DOI:** 10.3390/v15030750

**Published:** 2023-03-14

**Authors:** Zhangfu Chen, Haiyu Zhao, Zhongkuan Li, Mengkun Huang, Nan Si, Hui Zhao, Xiaolu Wei, Bo Sun, George F. Gao, Ziqian Xu, William J. Liu

**Affiliations:** 1 School of Public Health, Shandong University, Jinan 250012, China; 2 NHC Key Laboratory of Biosafety, National Institute for Viral Disease Control and Prevention, Chinese Center for Disease Control and Prevention (China CDC), Beijing 102206, China; 3 Research Unit of Adaptive Evolution and Control of Emerging Viruses (2018RU009), Chinese Academy of Medical Sciences, Beijing 102206, China; 4 Institute of Chinese Materia Medica, China Academy of Chinese Medical Sciences, Beijing 100700, China; 5 Institute of Special Animal and Plant Sciences, Chinese Academy of Agricultural Sciences, Changchun 130112, China; 6 Guangxi Key Laboratory of AIDS Prevention and Treatment, School of Public Health, Guangxi Medical University, Nanning 530000, China; 7 CAS Key Laboratory of Pathogen Microbiology and Immunology, Institute of Microbiology, Chinese Academy of Sciences (CAS), Beijing 100101, China

**Keywords:** phenuiviruses, bastrovirus, RNA virome, metagenomics sequencing, Asiatic toad, amphibian

## Abstract

Most zoonotic pathogens originate from mammals and avians, but viral diversity and related biosafety risk assessment in lower vertebrates also need to be explored. Amphibians are an important group of lower vertebrates that played a momentous role in animal evolution. To elucidate the diversity of RNA viruses in one important species of amphibians, the Asiatic toad (*Bufo gargarizans*), we obtained 44 samples including lung, gut, liver, and kidney tissues from Asiatic toads in Sichuan and Jilin provinces, China, for viral metagenomics sequencing. More than 20 novel RNA viruses derived from the order *Bunyavirales* and 7 families of *Astroviridae*, *Dicistroviridae*, *Leviviridae*, *Partitiviridae*, *Picornaviridae*, *Rhabdoviridae*, and *Virgaviridae* were discovered, which were distinct from previously described viruses and formed new clusters, as revealed by phylogenetic analyses. Notably, a novel bastrovirus, AtBastV/GCCDC11/2022, of the family *Astroviridae* was identified from the gut library, the genome of which contains three open reading frames, with the RNA-dependent RNA polymerase (RdRp) coded by ORF1 closely related to that of hepeviruses, and ORF2 encoding an astrovirus-related capsid protein. Notably, phenuiviruses were discovered for the first time in amphibians. AtPhenV1/GCCDC12/2022 and AtPhenV2/GCCDC13/2022 clustered together and formed a clade with the group of phenuiviruses identified from rodents. Picornaviruses and several invertebrate RNA viruses were also detected. These findings improve our understanding of the high RNA viral diversity in the Asiatic toad and provide new insights in the evolution of RNA viruses in amphibians.

## 1. Introduction

Emerging infectious diseases (EIDs), such as SARS and COVID-19, occur frequently worldwide [1,2]. The majority of EIDs are zoonotic, with over 70% caused by pathogens of wildlife origin [3], and pose a serious threat to human health and the economy [4]. In order to detect and prevent pandemics of emerging viral infections, active surveillance of viruses carried within wild animals has been intensified globally [5,6]. The development of metagenomics and next-generation sequencing has revolutionized the way in which we discover and explore viruses [7,8,9,10,11].

Lower vertebrates, comprising fish, amphibians, and reptiles, represent important host groups whose viral diversity is far less known and recently has been expanded by metagenomics [12,13,14], in addition to that of mammals such as bats [15,16] and rodents [17]. As the first terrestrial vertebrates, amphibians live in a special ecological environment and play a very important role in animal evolution and the ecological equilibrium [18]. Notably, various RNA viruses have been discovered in amphibians through metagenomics method in recent years. For example, the novel influenza-like virus identified in Wuhan Asiatic toad forms a sister group to that of influenza B virus, and the virus found in Ornate chorus frog clusters with influenza D virus [13,19]. In addition, new nidoviruses and hepadnaviruses were also identified in amphibians, expanding the host range of these viruses and shedding light on the biorisks posed by these lower vertebrates [20,21,22].

The Asiatic toad (*Bufo gargarizans*) belongs to the family of *Bufonidae* (Amphibia: Anura) and is widely distributed in East Asia [23]. The Asiatic toad is widely farmed to produce Chansu, which is a traditional Chinese medicine derived from the dried secretions and parotid glands from the toad’s skin [24]. The principal bioactive components, such as bufalin (BFL) and cinobufagin (CBG), are extracted from Chansu and have long been used as anticancer agents in China and other Asian countries [25].

In this study, we present an overview of the RNA viromes of Asiatic toads collected from two sites in China and characterized the novel RNA viruses, including a bastrovirus, bunyaviruses, a rhabdovirus, picornaviruses, and other invertebrate viruses. This is the first report of viral diversity in the Asiatic toad by metagenomics sequencing, expands the host range of various viruses and provides important insights into the understanding of the evolution of RNA viruses in amphibians.

## 2. Materials and Methods

### 2.1. Sample Collection

In April 2022, nine semi-artificial breeding Asiatic toads were sampled from Sichuan province in China, obtaining four types of tissue from each individual, including lung, gut, liver, and kidney. In July 2022, eight semi-artificial breeding toads were collected from Jilin province in China, and the lung tissue was obtained. The host species were first determined temporarily by morphology at the time of collection, after which the assembled scaffolds obtained by sequencing were compared with the local cytochrome *c* oxidase (COI) database to further confirm species classification. All samples were immediately transported in dry ice to the laboratory in Beijing and then stored at −80 °C for later RNA extraction.

### 2.2. RNA Extraction

Firstly, the samples were washed with 1×PBS buffer (Solarbio, Beijing, China), and then approximately 100 mg of tissue was transferred to an extraction tube (NZK biotech, Hubei, China) containing 1 mL of DMEM basal medium (Gibco, Grand Island, NY, USA) with 1% penicillin–streptomycin and homogenized at 60 Hz for 5 min using a Frozen Grinder (Shanghai Jingxin, Shanghai, China). Clear supernatants were obtained after centrifugation at 12,000 rpm for 10 min at 4 °C. According to the site of sample collection and type of tissue, the supernatants from each sample were pooled for library preparation. A total of six libraries were generated, each of approximately 1 mL, consisting of 1–9 samples. Total RNA was extracted from 200 μL of the mixed supernatants of each library using TRIzol (Ambion, Austin, TX, USA) and quantified by a Qubit Fluorometer (Invitrogen, Carlsbad, CA, USA) and Qubit RNA High-Sensitivity (HS) Assay Kit (Invitrogen, Carlsbad, CA, USA) for further sequencing.

### 2.3. RNA Library Construction and Sequencing

Single-stranded circular DNA libraries were constructed using the MGIEasy RNA Library preparation reagent set (MGI, Shenzhen, China) with 100–200 ng of total RNA input per library. Briefly, RNA was first fragmented and then converted to cDNA. The remaining cDNA was converted to double-stranded DNA and then subjected to purification, end-repair, A-tailing, adaptor ligation, and amplification. Finally, 60 fmol of PCR products were circularized and amplified by rolling-circle replication (RCR) to generate DNA nanoball (DNBs)-based libraries. The libraries were sequenced on a MGISEQ-200RS system (MGI, Shenzhen, China) sequencing platform with the 100 bp paired-end method.

### 2.4. Virus Discovery

During the quality control of the raw data, the reads containing adaptor sequences, with low sequencing quality and high content of N bases, were removed to obtain clean reads. For each library, the clean reads were assembled de novo using the Microbial Genome Analysis Pipeline (MGAP) with default parameter settings to obtain scaffolds. To confirm the viral scaffolds, the assembled scaffolds were compared by BLASTx analysis with the database comprising the RNA-dependent RNA polymerase (RdRp) protein of RNA viruses downloaded from GenBank [19,26]. The E-value cut-off for these comparisons was set at 1 × 10^−5^. To exclude false positives, the putative viral sequences showing similarity to the host, plant, bacterial, and fungal sequences were manually rejected by online BLASTx analysis against the NCBI non-redundant protein database (NR). Subsequently, the remaining scaffolds with unassembled overlaps classified as the same virus order or family were extracted and assembled with the SeqMan program (DNAstar v7.1). According to the results of the BLASTx analyses described above, viral scaffolds sharing <90% of RdRp amino acid identity with previous known viruses were identified as new viruses [27].

In order to fill the gaps in the viral genomes, we designed several sets of primers for RT-PCR or performed re-sequencing (using the metagenomics approach described above) od the individual samples that contained the target virus, to obtain longer viral genomic sequences. The viral genome sequences were used as reference genomes for reads mapping using MGAP to determine the reads abundances, and the final consensus sequences were determined from the final assembly of the mapped reads using IGV v2.11.9.

### 2.5. Genomic and Phylogenetic Analysis

The potential open reading frames (ORFs) of the viral genomes were predicted with the NCBI ORF Finder (https://www.ncbi.nlm.nih.gov/orffinder/, accessed on 11 February 2023). The putative conserved functional domains of the encoded proteins were annotated by the NCBI conserved domain database (CDD) (https://www.ncbi.nlm.nih.gov/cdd/, accessed on 11 February 2023). Pairwise alignments were performed by MegAlign (DNAstar v7.1). Putative viral genomes and their relative members were aligned by multiple alignment using MAFFT v7.0 and the ClustalW program (MEGA v7.0). All reference viral sequences were downloaded from GenBank. Phylogenetic trees were constructed using the Maximum Likelihood method in PhyML v3.1 with 1000 bootstrap replicates.

### 2.6. Viral Prevalence Calculation

In order to determine the presence of viruses in individual samples per each library, specific primers were designed for RT-PCR. Afterward, the target PCR products were confirmed by agarose gel electrophoresis and then subjected to Sanger sequencing (Beijing Tianyi-Huiyuan Biotechnology Co., Ltd., Beijing, China).

### 2.7. Virus Isolation

The positive samples determined using specific primers were inoculated into the African clawed frog kidney cell line ACTK-1 for virus isolation. The cells were cultured in 24-well cell culture plates with DMEM basal medium containing 10% fetal bovine serum and 1% penicillin–streptomycin in an incubator containing 5% carbon dioxide at 28 °C for more than 12 h. The homogenate supernatant was inoculated when the monolayer cell culture was about 90% confluent and adherent to the plate. The medium used was DMEM basal medium containing 2% fetal bovine serum. Three blind passages were performed for each sample. The growth and morphological changes of the cells were observed under a microscope every day.

## 3. Results

### 3.1. Sampling Results

A total of 44 samples from 17 individuals of Asiatic toad were collected from Sichuan and Jilin provinces in our study. Nine semi-artificial breeding toads were collected from Sichuan province, and four types of tissue including lung, gut, liver, and kidney were obtained. Eight semi-artificial breeding toads were collected from Jilin province, and the lung tissues were obtained. These samples were divided into six libraries based on the site of sampling and type of tissue, with one to nine samples in each library (Table 1).

### 3.2. Overview of RNA Viromes in Asiatic Toad

**Figure 1:**
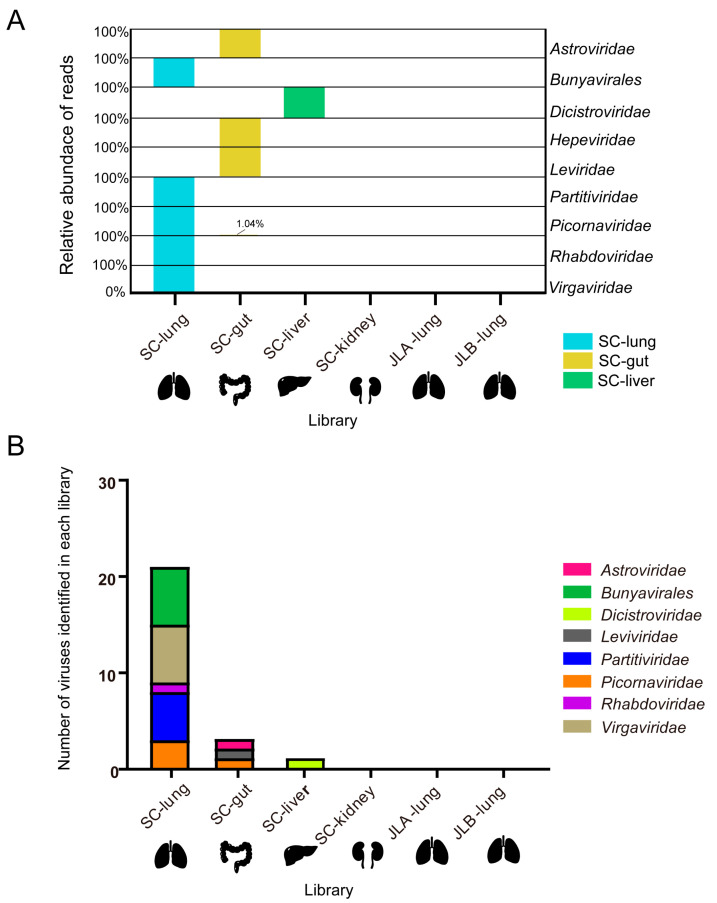
Relative abundance of normalized reads mapping to virus order/families and overview of the RNA viruses identified in each library. (**A**) Relative abundance of normalized viral reads mapping to the viral scaffolds of each virus order/families within each library, falling across nine viral order/families. The smallest reads in each virus order/family are defined as 0%, and the largest reads are defined as 100%; the result of the normalized reads is presented as a percentage. (**B**) Distribution of the number of viruses in each library.

A total of 78.2 GB of nucleotide data (782,063,744 raw reads, PE100) were obtained after viral metagenomics sequencing of six libraries, with 9~17 GB per library, and the Q30 of each library was more than 89%. After de novo assembly of 769,603,378 clean reads, 2244 scaffolds (> 500 bp) were obtained (Table 1). After the BLASTx analyses, 37 scaffolds were identified as viral scaffolds from three libraries (SC-lung, SC-gut, and SC-liver), which were collected from Sichuan province. These viral scaffolds were further identified and classified into the order *Bunyavirales* and into eight families, i.e., *Astroviridae*, *Dicistroviridae*, *Hepeviridae*, *Leviviridae*, *Partitiviridae*, *Picornaviridae*, *Rhabdoviridae*, and *Virgaviridae*. To obtain the relative abundance of reads in each viral order/family, the reads were mapped back to the viral scaffolds (Figure 1A). The mapping results revealed that the family *Picornaviridae* was present in both of the gut and the lung libraries. Viral reads from the order *Bunyavirales* and the families *Partitiviridae*, *Rhabdoviridae*, and *Virgaviridae* were only identified in the lung library. Viral reads from the families *Hepeviridae*, *Astroviridae*, and *Leviviridae* were only found in the gut library, and the family *Dicistroviridae* was only in the liver library. Thus, the distribution of these viruses in different tissue appears to be distinct, suggesting a unique tissue tropism. No scaffolds related to RNA viruses were extracted from the assembled scaffolds in the JLA-lung and JLB-lung from Jilin province.

### 3.3. Characterization of Novel RNA Viruses in the Asiatic Toad

A total of 23 viruses were obtained after the assembly of the scaffolds, of which 22 were novel viruses, different from known viruses (Table 2). A total of 20 viruses belonging to five families (*Astroviridae*, *Partitiviridae*, *Picornaviridae*, *Rhabdoviridae*, and *Virgaviridae*) were found in the SC-lung library, with high viral diversity (Figure 1B). Several important novel RNA viruses were further characterized in this study.

#### 3.3.1. Bastrovirus

The sequencing scaffolds corresponding to the *Hepeviridae* and *Astroviridae* families in the SC-gut library were used to obtain a longer sequence by designing sets of primers. RT-PCR screening of all individual gut samples showed that seven of the nine samples (77.8%) were positive using the screening primer (AT-bas-F/R) (Table S1). To obtain the complete viral genome, metagenomics sequencing was performed for a randomly selected positive gut sample (SC-gut-9) again, and a nearly complete genome sequence (6994 nt), named Asiatic toad bastrovirus strain AtBastV/GCCDC11/2022, was assembled finally.

**Figure 2:**
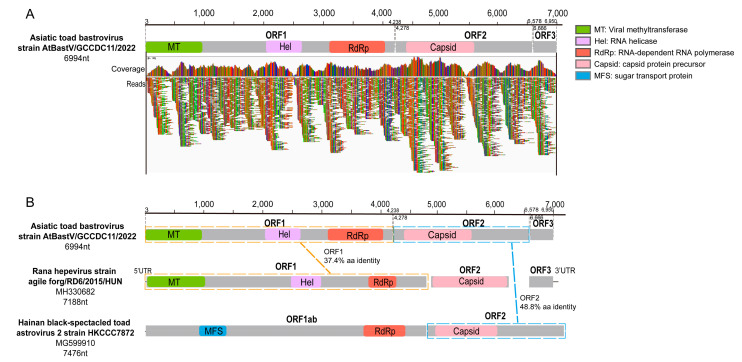
Schematic genome organization and comparisons of AtBastV/GCCDC11 with relative viruses. (**A**) AtBastV/GCCDC11 genomic organization consisting of three open reading frames (ORFs). Coverage and reads mapping to the genome are shown below the genome diagram. The genome map and each predicted ORF are drawn to scale. ORFs are shown in gray, and conservative functional domains are indicated in the legend on the right. (**B**) Comparisons of the genome structure of AtBastV/GCCDC11 with those of Rana hepevirus strain agile frog/RD6/2015/HUN (NC_040835) and Hainan black-spectacled toad astrovirus 2 (MG599910). The homology of ORF1 and ORF2 with other viruses is shown in the diagram.

According to ORFfinder (https://www.ncbi.nlm.nih.gov/orffinder/, accessed on 11 February 2023), the genome of AtBastV/GCCDC11 encodes three predicted ORFs. ORF1 encodes a non-structural protein of 1411 amino acids (aa) (4236 nt) containing viral methyltransferase, viral helicase, and RdRp domains (Figure 2A; Table S2). The BLASTp analyses revealed that the protein encoded by ORF1 shares an identity of 37.4% (E-value: 1 × 10^−134^, Query cover: 86%) with the corresponding non-structural protein of Rana hepevirus strain agile frog/RD6/2015/HUN (NC_040835) (Figure 2B). ORF2 encodes a capsid protein of 766 aa (2301 nt) which is similar to the astrovirus capsid protein precursor domain (Figure 2A; Table S2). Based on the BLASTp analyses, the protein encoded by ORF2 shares 48.8% identity with the capsid protein of Hainan black-spectacled toad astrovirus 2 (MG599910) (Figure 2B). ORF3, with unknown function, encodes a potential protein of 94 aa (285 nt), and no similar protein sequence was found in the GenBank database using BLAST searches (Figure 2A).

**Figure 3:**
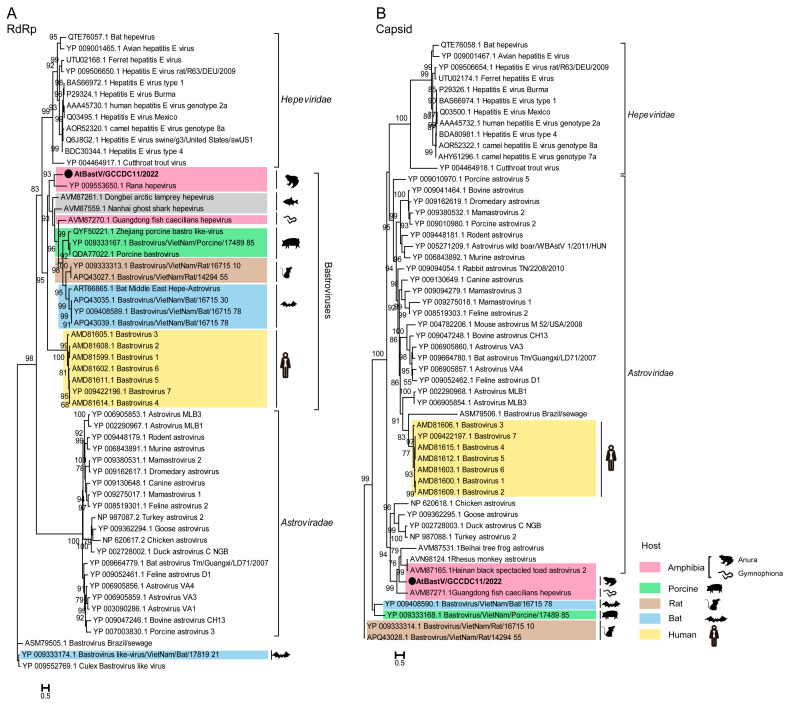
Phylogenetic analyses of AtBastV/GCCDC11 with other BastVs, astroviruses, and hepeviruses based on the amino acid sequences of RNA-dependent RNA polymerase (RdRp) and capsid domains. (**A**) Maximum Likelihood phylogenetic tree based on the RdRp domain, showing the RdRp coded by ORF1 closely related to that of hepeviruses. (**B**) Maximum Likelihood phylogenetic tree based on the capsid protein indicating that AtBastV/GCCDC11 branches with animal astroviruses and distantly relates to hepeviruses. AtBastV/GCCDC11 is marked with a black solid circle and indicated by bold text. Different hosts are marked in different colors. Percent bootstrap support is indicated by the value at each node. All trees are midpoint rooted, and taxonomy information is reported on the right of each tree.

The complete amino acid sequence of ORF1 and ORF2 from AtBastV/GCCDC11 was aligned with BastVs from amphibians, fish, swines, bats, rats, and humans. The ORF1 protein of AtBastV/GCCDC11 was found to share 28.5–34.3%, 29.7–31.2%, 31.5%, 30.7%, 30.8–31.3%, and 24.6–25.2% identity with amphibian, fish, porcine, bat, rat, and human BastVs, respectively (Table S3). The ORF2 protein of AtBastV/GCCDC11 showed 12.6–28.1%, 15.6%, 17.8%, 19.2%, and 19.5–20.1% identity with amphibian, porcine, bat, rat, and human BastVs, respectively (Table S4). Phylogenetic analyses based on the putative RdRp domain coded by ORF1 and the capsid protein domain coded by ORF2 of AtBastV/GCCDC11 were preformed using the Maximum Likelihood method with respect to other BastVs, astroviruses, and hepeviruses. The phylogenetic tree of RdRp showed that AtBastV/GCCDC11/2022 had the closest genetic relationship with the amphibian bastrovirus strain Rana hepevirus. AtBastV/GCCDC11/2022 branched with amphibian, porcine, rat, and bat BastVs, which independently formed clusters according to the host species. All BastVs and the hepatitis E virus formed a unique clade distinct from that of astroviruses (Figure 3A). The capsid phylogenetic tree showed that most animal BastVs formed a monophyletic clade and clustered by each host again. AtBastV/GCCDC11/2022 was closely related to Hainan black-spectacled toad astrovirus 2, and formed a clade with some animal astroviruses just like human BastVs, which branched with mammal astroviruses and was distantly related to hepeviruses (Figure 3B).

We attempted to isolate AtBastV/GCCDC11/2022 in ACTK-1 cells. Homogenates from the seven positive samples were inoculated in cell monolayers, and the supernatant of the infected cells was serially passaged three times, but no CPE was observed.

#### 3.3.2. Bunyaviruses

**Figure 4:**
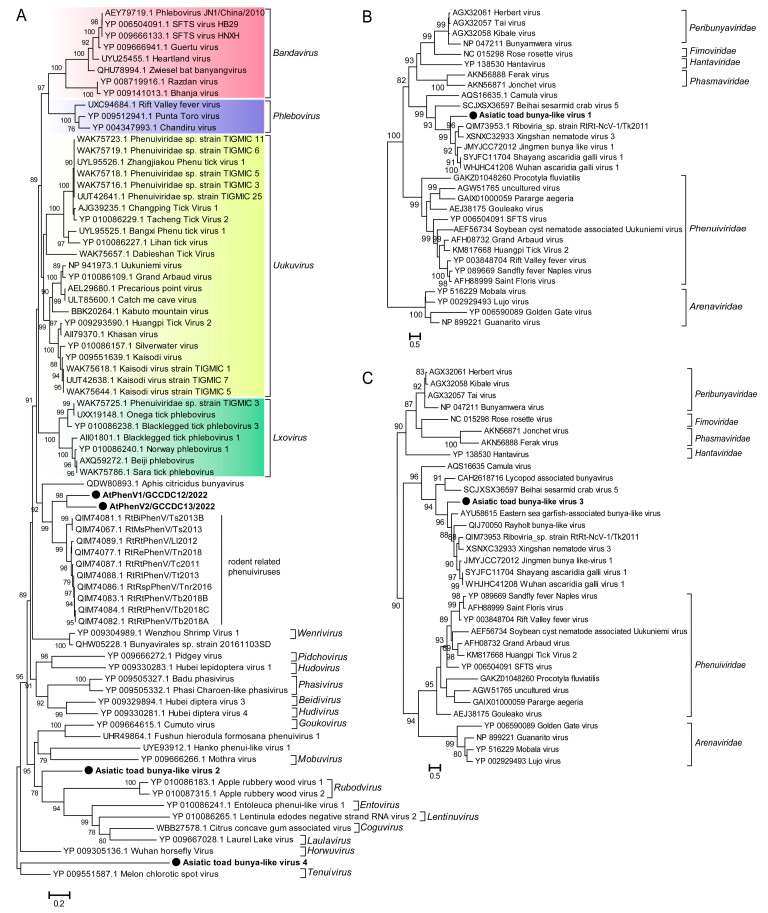
Phylogenetic analyses based on the RdRp sequences of representative viruses in the family *Phenuiviridae* and the order *Bunyavirales*. (**A**) Maximum Likelihood phylogenetic tree based on the RdRp proteins of novel phenui-like viruses with other representative viruses in the family *Phenuiviridae*, showing the position of these novel viruses within the diverse family *Phenuiviridae*. The novel phenui-like viruses identified in this study are marked with black solid circles and indicated by bold text. Lineages corresponding to the genera *Bandavirus*, *Phlebovirus*, *Uukuvirus*, and *Lxovirus* are colored red, purple, yellow, and green. (**B**,**C**) Maximum Likelihood phylogenetic tree based on the RdRp proteins of AtBunyV1 and AtBunyV3 with other viruses in the order *Bunyavirales*. The two bunya-like viruses are marked with black solid circles and indicated by bold text. Percent bootstrap support is indicated by the value at each node. All trees are midpoint rooted, and taxonomy information is reported on the right of each tree.

Partial genomic L segments encoding the RdRp domain of six novel bunya-like viruses were identified in the SC-lung library of the Asiatic toad, which were named Asiatic toad phenui-like virus strains AtPhenV1/GCCDC12/2022 and AtPhenV2/GCCDC13/2022 and Asiatic toad bunya-like virus strains AtBunyV1, AtBunyV2, AtBunyV3, and AtBunyV4 (Table 2). The BLASTp analyses based on the RdRp protein revealed that AtPhenV1/GCCDC12, AtPhenV2/GCCDC13, AtBunyV2, and AtBunyV4 were associated with virus members belonging to the family *Phenuiviridae* of the order *Bunyavirales* (Table 3). AtPhenV1/GCCDC12 and AtPhenV2/GCCDC13 exhibited 41.6% and 40.1% aa identity with the strains RtRt-PhenV/Tb2018B (MT085326) and RtRt-PhenV/Tc2011 (MT085330), respectively, which were identified in lung pools of rodents [28]. AtBunyV2 and AtBunyV4 were shown to share 28.8–30.6% aa identity with uukuviruses of the family *Phenuiviridae*, which were detected in the tick metagenome. AtBunyV1 and AtBunyV3 shared 42.6% and 40.8% aa identity with unclassified bunyaviruses identified from common pheasant gut roundworm and fish, respectively (Table 3). A phylogenetic tree constructed based on the RdRp sequences of representative viruses in the family *Phenuiviridae* suggested that the four novel phenuiviruses were located in different positions of the tree. The cluster of AtPhenV1/GCCDC12 and AtPhenV2/GCCDC13, which share 46.1% nucleotide identity with each other, appeared to form a branch with the lineage of rodent PhenVs previously described in rodent lungs and to fall at the basis of the genera *Bandavirus*, *Phlebovirus*, *Uukuvirus*, and *Lxovirus* (Figure 4A). AtBunyV2 and AtBunyV4 were also found to be located in a distinct clade of the family *Phenuiviridae* from the other known genera (Figure 4A). The RdRp tree of representative viruses in the order *Bunyavirales* showed that AtBunyV1 and AtBunyV3 fall within the clade of unclassified bunyaviruses (Figure 4B,C).

RT-PCR screening showed that 8/9 (88.9%) of AtPhenV1/GCCDC12/2022 and 4/9 (44.4%) of AtPhenV2/GCCDC13/2022 were positive using the screening primers AT-PHV1-F/R and AT-PHV2-F/R (Table S1), respectively. We attempted to isolate AtPhenVs from ACTK-1 cells, but no CPE was observed after three passages.

#### 3.3.3. Rhabdovirus

**Figure 5:**
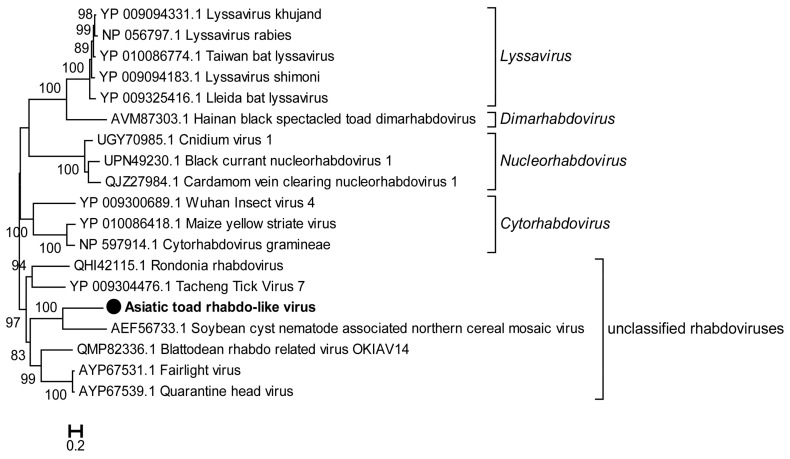
Phylogenetic analysis based on the amino acid sequence of the RdRp of AtRhabV compared with those of other rhabdoviruses. The tree was constructed using the Maximum Likelihood method in PhyML with 1000 bootstrap replicates. The novel strain identified in this study is marked with a black solid circle and indicated by bold text. Percent bootstrap support is indicated by the value at each node. Taxonomy information is reported on the right of each tree.

A novel rhabdovirus was also detected in the library SC-lung, which was tentatively called Asiatic toad rhabdo-like virus (AtRhabV). The genome of AtRhabV (5068nt) encodes a protein of 1688 aa containing the RdRp domain, sharing 32.1% aa identity with the corresponding protein of Soybean cyst nematode-associated northern cereal mosaic virus (ScRV) (HM849039) (Table 2). A phylogenetic tree based on the RdRp protein sequences of AtRhabV and other rhabdoviruses (RhabVs) revealed that AtRhabV independently formed a cluster with ScRV of the genus *Cytorhabdovirus* and branched with other unclassified RhabVs (Figure 5).

#### 3.3.4. Picornaviruses and Other Invertebrate RNA Viruses

**Figure 6:**
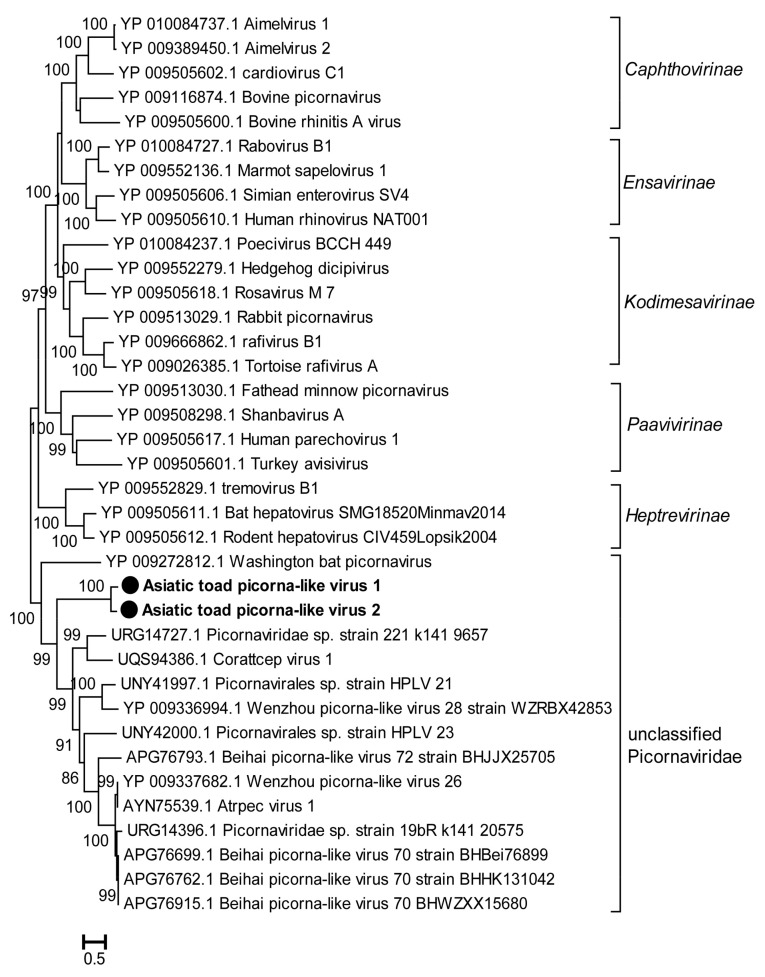
Phylogenetic analysis based on the amino acid sequence of the RdRp of picornaviruses. The phylogenetic tree was constructed using the Maximum Likelihood method in PhyML with 1000 bootstrap replicates. The novel strains identified in this study are marked with black solid circles and indicated by bold text. Percent bootstrap support is indicated by the value at each node. Taxonomy information is reported on the right of each tree.

A total of three novel picornaviruses (PicoVs) were detected in the lung and gut tissues. The virus called Asiatic toad picorna-like virus 1 (AtPicoV1) was identified in both lung and gut samples. Due to the incomplete genome of AtPicoV3, the RdRp domain was not predicted, so we constructed a phylogenetic tree based on the amino acid sequence of RdRp in AtPicoV1 and AtPicoV2. Phylogenetic analysis indicated that AtPicoV1 and AtPicoV2 formed a new cluster within a clade of unclassified PicoVs (Figure 6). In addition to the novel viruses described in the above results, we also detected several invertebrate RNA viruses of the families of *Dicistroviridae* (*n* = 1), *Leviviridae* (*n* = 1), *Partitiviridae* (*n* = 4), and *Virgaviridae* (*n* = 6) (Table 2). Among these invertebrate viruses, except for Wuhan spider virus 10, 11 viruses were distantly related to the known viruses and exhibited 27.2–51.8% aa identity with their closest relatives (Table 2), indicating that these viruses may belong to new genera or families.

## 4. Discussion

Amphibians are a quite important group of lower vertebrates that live in a special ecological environment and play a momentous role in the process of animal evolution [18]. As the source of Chansu, a precious traditional Chinese medicine, Asiatic toads have been widely farmed in many Asian countries [23,25]. Interestingly, a meta-transcriptomic analysis performed in fish, amphibians, and reptiles revealed a novel influenza-like virus in Wuhan Asiatic toad [19]. Notably, various RNA viruses have been identified in amphibians through metagenomics sequencing [20,21,22]. Therefore, we selected the Asiatic toad as the study object to further explore the RNA viral diversity. We identified 23 RNA viruses from the lung, gut, and liver of Asiatic toads collected in Sichuan province. No viruses were discovered in Asiatic toads collected in Jilin province, possibly due to the limited number of samples or to regional differences. The findings of this study demonstrated that the Asiatic toad harbors a wide diversity of RNA viruses belonging to the order *Bunyavirales* and to the seven families of *Astroviridae*, *Dicistroviridae*, *Leviviridae*, *Partitiviridae*, *Picornaviridae*, *Rhabdoviridae*, and *Virgaviridae*.

A novel bastrovirus called AtBastV/GCCDC11/2022 was found in the gut samples of Asiatic toads, with similar genomic characteristics to those previously described for BastVs. BastVs is a novel group of hepe-astrovirus-like viruses identified in human stools [29], raw sewage [30], pigs [31], bats [32], rats, as well as in clams [33] by metagenomics sequencing. The RNA genome of AtBastV/GCCDC11 consists of three ORFs: ORF1, ORF2, and ORF3. ORF1 encodes a putative non-structural protein containing three predicted conserved domains of viral methyltransferase, RNA helicase, and RdRp, whereas ORF2 encodes the astrovirus capsid protein. ORF3 encodes a putative protein with unknown function. A phylogenetic analysis revealed that ORF1 shares identity with the non-structural domains of the family *Hepeviridae* and is a close relative of the Rana hepevirus, which was detected in the lung of agile frogs (*Rana dalmatina*), with five domains being observed in ORF1, a capsid protein encoded by ORF2, and a putative protein with unknown function encoded by ORF3 [34]. The phylogenetic tree indicated that the capsid protein encoded by ORF2 is closely related to the capsid protein of the family *Astroviridae*. Thus, the RdRp and the capsid regions of AtBastV/GCCDC11 were more phylogenetically related to those of hepatitis E viruses and astroviruses, respectively, providing further evidence that BastVs may have originated from recombination between hepeviruses and astroviruses in ancient times [29]. Furthermore, the observation that AtBastV/GCCDC11 branched with amphibians BastVs both in the RdRp and in the capsid trees and the high prevalence of AtBastV/GCCDC11 in the individuals suggested that amphibian BastVs may have evolved over a long period in amphibian populations.

The order *Bunyavirales* contains important pathogens that can cause severe diseases in humans and animals, such as Crimean–Congo hemorrhagic fever virus of the family *Nairoviridae*, SFTS virus, Toscana virus, and Rift Valley fever virus of the family *Phenuiviridae* [35,36,37,38], whose genomes are negative single-stranded RNAs consisting of Large (L), Medium (M), and Small (S) segments [39].We also identified six bunya-like viruses in our study, four of them belonging to the family *Phenuiviridae*. This is the first identification of phenuiviruses in amphibians. The phylogenetic analysis revealed that the cluster of the two strains AtPhenV1/GCCDC12 and AtPhenV2/GCCDC13 was related to that of rodent PhenVs detected in the lung samples [28]; AtBunyV2 and AtBunyV4 formed separate clades, AtBunyV1 and AtBunyV3 both belonged to the unclassified bunyaviruses. These novel amphibian bunya-like viruses may represent novel genera or families. The long branch between the AtPhenV1/GCCDC12 and AtPhenV2/GCCDC13 indicated that there may be various amphibians PhenVs yet to be discovered.

In the lung samples, a novel rhabdovirus was also identified. RhabVs are a large and ecologically diverse group of negative-sense, single-stranded RNA viruses in the family *Rhabdoviridae* of the order *Mononegavirales*, which infect vertebrates, invertebrates, and plants [40,41]. The majority of RhabVs are transmitted by arthropods to vertebrate or plant hosts, but some of them have evolved to circulate among vertebrates without a biological vector and can cause mild-to-severe diseases such as vesicular stomatitis virus and rabies virus [42]. The rhabdovirus previously identified in Hainan black-spectacled toad belonged to the genus *dimarhabdovirus*, which was associated with vector-borne transmission [19], whereas the novel rhabdovirus identified in the Asiatic toad in our study showed a low amino acid homology with known *Rhabdoviridae* members. The most closely related virus appeared to be the Soybean cyst nematode-associated northern cereal mosaic virus (ScRV), a newly identified RhabV in soybean cyst nematode [43]. Considering that amphibians are frequently infected by parasites [44], which differ in species in different organs and tissues, and nematodes are mainly found in the lungs and intestine [45], we cannot rule out the possibility that the above RhabV could have originated from parasites in the toads.

In addition, diverse PicoVs were detected in the lung and gut samples and formed a distinct cluster based on the phylogenetic tree of RdRp protein sequences. Members of the family *Picornaviridae* are small, nonenveloped, positive single-stranded RNA viruses that can cause a variety of human diseases such as poliomyelitis, common cold, myocarditis, and hepatitis [46]. Many PicoVs infecting amphibians have been discovered by sequencing over the years [19,47,48,49,50]. The identification of AtPicoVs will help to expand our knowledge of the family *Picornaviridae*, which will facilitate the study of amphibian PicoVs in the future. On the other hand, thousands of novel viral genomes from invertebrate samples have recently identified by meta-transcriptomic studies [47,51,52]. In our study, we identified several invertebrate RNA viruses from three libraries (SC-liver, SC-lung, and SC-gut) derived from liver, lung, and gut samples.

Our study enriches the diversity of RNA viruses in the Asiatic toad, expands the host range of the known virus families, and suggests that amphibians should be considered as potential carriers and transmitters of these viruses. However, there are also shortcomings in our study, such as the limited number of samples and the fact that only lung specimens were collected from Asian toads in Jilin Province. In future studies, the sample size will be further increased and other tissues will be obtained for sequencing.

## 5. Conclusions

In conclusion, the present study provides an overview of the RNA virome in the Asiatic toad. We discovered 22 novel RNA viruses of 8 different order/families in the Asiatic toad, including a novel bastrovirus whose nearly complete genome showed potential intermediate features between those of hepeviruses and astroviruses and the first identified phenuiviruses in amphibians. These findings highlight the wide diversity of RNA viruses in the Asiatic toad and increase our understanding of the evolution and potential cross-species transmission of viruses in amphibians.

## Figures and Tables

**Table 1:** Information on the sample libraries and sequencing data results.

Site	Date of Collection (mm/yyyy)	Individual Number	Tissue (Number)	Library	Raw Reads	Clean Reads	Clean Base Q30 ^1^	Scaffolds Number	Scaffold Average Length (bp)	N50 ^2^
Sichuan	4/2022	9	Lung (9)	SC-lung	172,714,428	171,489,864	89.7%	350	952.9	966
			Gut (9)	SC-gut	133,230,938	132,643,352	93.3%	811	987.8	1009
			Liver (9)	SC-liver	158,158,996	157,069,570	90.2%	809	962.2	937
			Kidney (9)	SC-kidney	127,191,896	126,644,714	92.8%	236	974.2	1011
Jilin	7/2022	8	Lung (3)	JLA-lung	94,277,790	89,464,230	93.1%	13	1290.4	1706
			Lung (5)	JLB-lung	96,489,696	92,291,648	93.3%	25	1582.4	1973

^1^ Q30 is defined as a property that is logarithmically related to the base calling error probabilities. It is equivalent to the probability of an incorrect base call 1 in 1000 times. This means that the base call accuracy (i.e., the probability of a correct base call) is 99.9%. ^2^ The N50 scaffolds set is calculated by summarizing the lengths of the biggest scaffolds until you reach 50% of the total scaffold length. The minimum scaffold length in this set is the number that is usually used to report the N50 value of a de novo assembly.

**Table 2:** Viral genomes identified in the Asiatic toad and BLASTx hits on known viruses.

Order/Family	Library	Virus Name	Length (bp)	BLASTx Hits on Known Viruses (Blast Amino Acid Identity)	Reads
*Astroviridae*	SC-gut	AtBastV/GCCDC11/2022	6994	Rana hepevirus (36.6%)	301
*Bunyavirales*	SC-lung	AtPhenV1/GCCDC12/2022	2270	*Phenuiviridae sp.* strain RtRt-PhenV/Tb2018B (41.6%)	82
		AtPhenV2/GCCDC13/2022	2622	*Phenuiviridae sp.* strain RtRt-PhenV/Tc2011 (40.1%)	142
		Asiatic toad bunya-like virus 1	769	Jingmen bunya-like virus 1 (42.6%)	27
		Asiatic toad bunya-like virus 2	1752	*Bunyavirales sp.* strain 20161103SD (26.0%)	109
		Asiatic toad bunya-like virus 3	573	Eastern sea garfish-associated bunya-like virus (40.8%)	20
		Asiatic toad bunya-like virus 4	781	*Phenuiviridae sp.* strain TIGMIC_25 (30.6%)	36
*Dicistroviridae*	SC-liver	Asiatic toad picorna-like virus 4	745	*Dicistroviridae sp.* strain XZN128099 (46.1%)	25
*Leviviridae*	SC-gut	Asiatic toad levi-like virus	581	ssRNA phage AVE015 (51.8%)	29
*Partitiviridae*	SC-lung	Wuhan spider virus 10 *	763	Wuhan spider virus 10 (95.2%)	35
		Asiatic toad partiti-like virus 1	1554	Xinzhou partiti-like virus 1 (44.8%)	297
		Asiatic toad partiti-like virus 2	1386	Xinzhou partiti-like virus 1 (51.7%)	231
		Asiatic toad partiti-like virus 3	865	Xinzhou partiti-like virus 1 (48.5%)	105
*Picornaviridae*	SC-lung	Asiatic toad picorna-like virus 1	9550	*Picornaviridae sp.* strain 19bR-k141_20575 (34.0%)	12,849
		Asiatic toad picorna-like virus 2	3539	Corattcep virus 1 (26.0%)	373
		Asiatic toad picorna-like virus 3	1013	*Cripavirus sp.* strain s59-k141_1227864 (35.1%)	94
	SC-gut	Asiatic toad picorna-like virus 1	735	Wenzhou picorna-like virus 26 (37.3%)	141
*Rhabdoviridae*	SC-lung	Asiatic toad rhabdo-like virus	5068	Soybean cyst nematode associated northern cereal mosaic virus (32.1%)	306
*Virgaviridae*	SC-lung	Asiatic toad virga-like virus 1	2144	Nelorpivirus dungfly (37.1%)	167
		Asiatic toad virga-like virus 2	575	Clonorsi virus 1 (29.0%)	46
		Asiatic toad virga-like virus 3	1288	Blueberry necrotic ring blotch virus (46.0%)	73
		Asiatic toad virga-like virus 4	930	Hubei negev-like virus 1 (38.8%)	60
		Asiatic toad virga-like virus 5	1091	Bemisia tabaci nege-like virus 1 (31.8%)	65
		Asiatic toad virga-like virus 6	1077	Fasciohepa virus 3 (27.2%)	82

* Previously known virus.

**Table 3:** Genomic characterization of bunyaviruses identified in the Asiatic toad and BLASTp hits on known viruses.

Virus Name	Length (nt)	Protein Size (aa)	BLASTp Hit (Host)	aa Identity
AtPhenV1/GCCDC12/2022	2270	756	*Phenuiviridae sp.* strain RtRt-PhenV/Tb2018B (*Rattus tanezum*)	41.6%
AtPhenV2/GCCDC13/2022	2622	873	*Phenuiviridae sp.* strain RtRt-PhenV/Tc2011 (*Rattus tanezum*)	40.4%
Asiatic toad bunya-like virus 1	769	256	Jingmen bunya-like virus 1 (common pheasant gut roundworm)	42.6%
Asiatic toad bunya-like virus 2	1752	583	*Kaisodi virus* strain TIGMIC_1 (*Haemaphysalis montgomeryi*)	28.8%
Asiatic toad bunya-like virus 3	573	190	Eastern sea garfish-associated bunya-like virus (*Hyporhamphus australis*)	40.8%
Asiatic toad bunya-like virus 4	781	259	*Phenuiviridae sp.* strain TIGMIC_25 (*Dermacentor nuttalli*)	30.6%

## Data Availability

All virus genome sequences generated in this study were deposited in GenBank under the accession numbers OQ599876–OQ599897. The raw reads were submitted to the Sequence Read Archive (SRA) under the project ID PRJNA936938.

## Supplementary Materials

The following supporting information can be downloaded at: https://www.mdpi.com/article/10.3390/v15030750/s1, Table S1: RT-PCR primers used to screen the prevalence of viruses in our study; Table S2: Conserved domains identified in the ORF1 and ORF2 of AtBastV; Table S3: Pairwise amino acid identities corresponding to ORF1 for BastVs; Table S4: Pairwise amino acid identities corresponding to ORF2 for BastVs.
