# Supplementary material for: First Discovery of Phenuiviruses within Diverse RNA Viromes of Asiatic Toad (Bufo gargarizans) by Metagenomics Sequencing
Source: Viruses. 2023 Mar 14;15(3):750. doi: 10.3390/v15030750 (PMC10056474; doi:10.3390/v15030750)
Supplement: Supplementary file 1 [file viruses-15-00750-s001.zip › viruses-2255612-supplementary.pdf]

Supplementary Table S1. RT-PCR Primers used to screen the prevalence of viruses in our study

Virus name	Primer name	Sequence (5'→3')	Length	Product length
AtBastV/GCCDC11/2022	AT-bas-F	TCCCGCAGAACAGTGACCATTT	22	368
	AT-bas-R	GCCATAACGTGCCTCATACCG	21	
AtPhenV1/GCCDC12/2022	AT-PHV1-F	CTACTTCCTGTGAGTCTTTCCCTA	24	321
	AT-PHV1-R	TTTGCAGTTCTTTCCGTTCC	20	
AtPhenV2/GCCDC13/2022	AT-PHV2-F	TGGGTTACTTAAAGGATAAGAACG	24	180
	AT-PHV2-R	ACTTGGTCAGGGAGGGAGAA	20	

Supplementary Table 2. Conserved domains identified in the ORF1 and ORF2 of AtBastV

ORF	Identified domain	Description	Start (aa)	End (aa)
ORF1	Vmethyltransf	Viral methyltransferase	39	345
ORF1	Viral_helicase1	Viral (Superfamily 1) RNA helicase	668	892
ORF1	RdRP_2	RNA dependent RNA polymerase	1049	1351
ORF2	Astro_capsid_N	Astrovirus capsid protein precursor	89	449

Supplementary Table 3. Pairwise amino acid identities corresponding to ORF1 for BastVs

		AtBastV	YP_009553650.1	AVM87270.1	AVM87261.1	AVM87559.1	YP_009333167.1	YP_009408589.1	APQ43027.1	YP_009333313.1	AMD81599.1	YP_009422196.1
Amphibian	AtBastV/GCCDC11/2022		34.3	28.5	31.2	29.7	31.5	30.7	30.8	31.3	25.2	24.6
	YP_009553650.1 Rana hepevirus			26.0	29.0	28.1	29.8	30.1	27.9	27.6	22.9	23.2
	AVM87270.1 Guangdong fish caecilians hepevirus				36.2	38.4	38.8	40.1	38.7	38.8	26.0	25.0
Fish	AVM87261.1 Dongbei arctic lamprey hepevirus					40.6	34.5	35.1	34.5	34.7	25.3	25.2
	AVM87559.1 Nanhai ghost shark hepevirus						36.0	36.5	37.6	38.0	25.5	25.5
Porcine	YP_009333167.1 VietNam/Porcine/17489_85							45.1	42.1	42.7	24.1	23.6
Bat	YP_009408589.1 VietNam/Bat/16715_78								49.2	50.0	25.9	25.3
Rat	APQ43027.1 VietNam/Rat/14294_55									94.6	24.4	24.3
	YP_009333313.1 VietNam/Rat/16715_10										25.1	25.3
Human	AMD81599.1 Bastrovirus 1											83.7
	YP_009422196.1 Bastrovirus 7											

Supplementary Table 4. Pairwise amino acid identities corresponding to ORF2 for BastVs

		AtBastV	YP_009553651.1	AVM87270.1	YP_009333168.1	YP_009408590.1	APQ43028.1	YP_009333314.1	AMD81600.1	YP_009422197.1
Amphibian	AtBastV/GCCDC11/2023		12.6	28.1	15.6	17.8	19.2	19.2	19.5	20.1
	YP_009553651.1 Rana hepevirus			13.5	10.3	14.2	13.2	13.4	9.6	10.8
	AVM87270.1 Guangdong fish caecilians hepevirus				15.8	18.5	15.8	15.6	19.3	19.5
Porcine	YP_009333168.1 VietNam/Porcine/17489_85					28.5	26.6	26.4	15.5	14.8
Bat	YP_009408590.1 VietNam/Bat/16715_78						35.5	35.5	18.2	17.8
Rat	APQ43028.1 VietNam/Rat/14294_55							93.0	19.4	18.3
	YP_009333314.1 VietNam/Rat/16715_10								19.8	18.8
Human	AMD81600.1 Bastrovirus 1									76.2
	YP_009422197.1 Bastrovirus 7									
